# Factors Affecting Consolidation in Geopolymers for Stabilization of Galvanic Sludge

**DOI:** 10.3390/ma18133015

**Published:** 2025-06-25

**Authors:** Francesco Genua, Mattia Giovini, Elisa Santoni, Mario Berrettoni, Isabella Lancellotti, Cristina Leonelli

**Affiliations:** 1 Department of Engineering “Enzo Ferrari”, University of Modena and Reggio Emilia, 41125 Modena, Italy; francesco.genua@unimore.it (F.G.); mattia.giovini@unimore.it (M.G.); cristina.leonelli@unimore.it (C.L.); 2 School of Science and Technology, University of Camerino, Camerino, 62032 Macerata, Italy; elisa.santoni@unicam.it (E.S.); mario.berrettoni@unicam.it (M.B.)

**Keywords:** alkali activation, stabilization, galvanic sludge, heavy metals, chromium, nickel, leaching, chemical stability

## Abstract

This study explores the potential of metakaolin-based geopolymers, activated using sodium hydroxide and sodium silicate, for the solidification and stabilization of heavy metals present in galvanic sludge—a hazardous industrial waste rich in chromium (Cr), nickel (Ni), and iron (Fe). The research investigates factors affecting the cold consolidation of the pastes, such as NaOH molarity (8 or 10 M) and time of preparation of activating solutions (24 h in advance or soon before the fresh paste preparation), the sequence of experimental steps (the sludge added to the fresh paste or to the powder of metakaolin) and amount of waste (10 or 20 per cent by weight over metakaolin). The final products were characterized by X-ray diffraction (XRD), Fourier-transform infrared spectroscopy (FT-IR), and environmental scanning electron microscopy (ESEM). Mechanical performance and durability assessments, including compressive strength and water stability tests, were conducted to evaluate the suitability of the geopolymer for construction applications. Leaching tests according to EU regulation demonstrated promising heavy metal immobilization, highlighting the effectiveness of the geopolymerization process in reducing metal leachability. It was found that the factors affecting immobilization are more evident for Cr than for Ni, whose immobilization percentages are very high. In particular, it was observed that preparing the mixture by adding sludge after metakaolin activation increased Cr immobilization from 83% to 89%. Similarly, preparing the activating solution 24 h before mixing the sludge and geopolymer increased the percentage from 89 to 95.

## 1. Introduction

Geopolymers have garnered significant attention as eco-friendly alternatives to traditional construction materials, particularly as sustainable substitutes for ordinary Portland cement (OPC) [1,2]. The increasing demand for environmentally sustainable construction materials is driven by the urgent need to reduce carbon emissions and the depletion of natural resources associated with conventional cement production, which is responsible for approximately 8% of global CO_2_ emissions [2].

Geopolymers are synthesized by combining an aluminosilicate precursor—such as fly ash, slag, or metakaolin—with an alkaline activator, typically sodium hydroxide (NaOH) and/or sodium silicate (Na_2_SiO_3_) [3]. The geopolymerization process involves a series of chemical reactions that result in the formation of a semi-crystalline, amorphous structure that exhibits properties analogous to those of hardened concrete. This reaction can take place at room temperature or under elevated temperatures, depending on the required properties of the final product [4].

Common sources of aluminosilicates for geopolymers include kaolin, metakaolin, and various industrial by-products such as fly ash, volcanic ash, granulated blast furnace slag, and other mineral wastes [5,6]. The utilization of these materials significantly contributes to reducing CO_2_ emissions associated with construction activities, achieving reductions of up to 80% compared to traditional OPC [2]. The successful incorporation of industrial by-products not only enhances the sustainability of geopolymers but also addresses the growing issue of waste disposal in the construction sector [7].

A pivotal aspect of the geopolymerization process is the dissolution of reactive alumina (Al_2_O_3_) and silica (SiO_2_) from the precursor material in an alkaline environment, leading to the formation of a robust three-dimensional network of Si-O-Al bonds [8]. The type and concentration of the alkaline activator play a crucial role in determining the properties of the geopolymer, including its setting time, compressive strength, and overall durability [9]. Furthermore, geopolymers exhibit excellent resistance to high temperatures and aggressive chemical environments, enhancing their appeal in a variety of industrial applications, including refractory materials and protective coatings [10].

One of the primary advantages of geopolymers is their capacity to encapsulate hazardous wastes, positioning them as ideal candidates for the stabilization/solidification (S/S) process [11,12]. The S/S process is a widely recognized technique for the treatment and disposal of hazardous materials, particularly those containing heavy metals, as it immobilizes contaminants within a stable matrix [13,14]. Traditionally, OPC has been the binder of choice for S/S applications due to its low cost and availability. However, OPC presents several drawbacks, including high energy consumption during production, significant CO_2_ emissions, and susceptibility to degradation in harsh environmental conditions [15]. In contrast, geopolymers have emerged as a promising alternative due to their superior mechanical properties, lower environmental impact, and enhanced resistance to chemical degradation [15]. Research has shown that geopolymers can effectively immobilize various hazardous substances, including heavy metals, radioactive elements, and organic pollutants, thus providing a multifaceted solution to waste management challenges [16,17,18].

Heavy metal contamination represents a significant environmental concern, primarily due to the persistence and toxicity of metals such as lead (Pb), mercury (Hg), cadmium (Cd), nickel (Ni), and chromium (Cr). These contaminants are often the by-products of various industrial activities, including mining, electroplating, and manufacturing, which lead to severe soil and water pollution that poses substantial risks to human health and ecosystems [19]. Heavy metals are known for their bioaccumulation potential, leading to toxic effects in living organisms, including endocrine disruption, neurotoxicity, and carcinogenicity [20]. Moreover, conventional remediation techniques, such as soil washing, thermal treatment, and chemical stabilization, often fail to effectively immobilize heavy metals in situ, underscoring the need for innovative approaches like geopolymerization to address this pressing issue [21].

The following section lists several real-world case studies that explore the stabilization of heavy metals in different systems through the use of geopolymer-based strategies.

**Treatment of Fly Ash from Municipal Solid Waste Incineration (MSWI):**

Fly ash generated from the incineration of municipal solid waste (MSWI-FA) often contains concentrations of hazardous heavy metals, including Pb, Cd, Cr (III), Cu, Ni, and Zn. Among these, Zn frequently exhibits the highest leaching values, up to 233.58 ppm in electrofilter ash, while Pb and Cu can also reach significant levels, with 60.80 ppm and 48.22 ppm, respectively [22]. These values often surpass regulatory limits, necessitating effective stabilization strategies to prevent environmental contamination. Geopolymers have demonstrated a strong capacity to immobilize these metals by incorporating MSWI-FA into aluminosilicate matrices, where the metals become physically encapsulated or chemically bound, thus minimizing their mobility [14,22,23,24].

In Lancellotti et al., a geopolymer system with 20% electrofilter fly ash (GPEF20) completely inhibited Pb and Cd leaching, while Cr (III) concentrations remained as low as 0.09 ppm, well under non-hazardous waste thresholds [14]. Another investigation using MSWI-FA reported a Pb leachate level of just 0.03 ppm, with Cd being undetectable, confirming the efficiency of this approach [24]. Similarly, when MSWI-FA was stabilized with 20% steel slag, Zn concentrations in leachates were reduced from 233.58 ppm (in untreated ash) to 6.469 ppm [24]. In the same system, Cr leaching dropped to 1.00 ppm from an initial 10.43 ppm, and Cu also showed strong reduction. These results collectively validate the use of geopolymers as a viable method for heavy metal stabilization in MSWI-FA, thanks to the alkaline synthesis conditions that facilitate hydroxide precipitation and encapsulation within the geopolymer network [14,23,24].

**Wastewater Treatment:**

Geopolymers synthesized from coal fly ash or metakaolin have also proven effective for removing heavy metals from aqueous solutions, particularly industrial wastewater. Fly ash-based geopolymers have shown high adsorption capacities; for instance, Pb removal reached 174.34 mg/g under optimized conditions (pH 5, 2-h contact time, and 1.5 g/L dosage) [25], while Cu adsorption reached 152 mg/g at 45 °C [25]. These materials have demonstrated consistent removal efficiency for common wastewater pollutants like Cu, Pb, and Cd, even when present in concentrations ranging from parts per billion to several mg/L.

Metakaolin-based geopolymers have also exhibited excellent adsorption characteristics, especially for Cu and Cr, due to their high surface area and structural stability upon alkali activation. Some systems achieved Cu removal up to 152 mg/g, and significant performance was observed for Pb and Cr as well [26]. Factors such as pH, temperature, and metal species influence the adsorption capacity, which typically follows Langmuir or Freundlich isotherms, indicating both monolayer and multilayer adsorption processes. Moreover, sodium-activated geopolymers generally exhibit higher retention of metal ions compared to potassium-based counterparts [25,26].

**Landfill and Soil Remediation:**

In the context of contaminated soils and landfills, geopolymers formulated with fly ash and ground granulated blast furnace slag (GGBS) have demonstrated substantial immobilization capabilities [27]. For instance, arsenic levels in untreated soils reached 234 µg/L, but treatment with alkali-activated slag drastically reduced its leachability, up to 92% within 28 days [27].

These improvements are closely tied to the increased content of binder materials (FA + GGBS) and curing duration, which enhance the mechanical strength of the treated matrix. In some cases, unconfined compressive strength (UCS) values reached up to 19 MPa [28,29]. Structural analyses revealed the formation of C-A-S-H and N-A-S-H gels, which contributed to a denser and more chemically resilient matrix. Moreover, pH-dependent leaching studies showed a significant drop in metal release at alkaline pH levels (~10), particularly for Cd [28,29].

**Nuclear Material Encapsulation:**

Geopolymers have gained attention as promising alternatives to cement for immobilizing radioactive waste. Cesium (Cs), known for its high solubility and mobility, was effectively stabilized in geopolymer matrices by exchange with Na, at concentrations between 0.5% and 2%, with leaching rates reduced to 0.5%, a stark improvement over traditional cement systems, where leaching often exceeds 30% [30]. Similarly, strontium (Sr), typically found at 1–3% in radioactive waste, was retained in the form of insoluble compounds such as Sr hydroxide or carbonate, achieving leaching rates below 3% [18].

The overall immobilization efficiency of these systems often exceeds 97%. Adjustments to the Si/Al ratio or the inclusion of additives like zeolites further improve performance. When processed at elevated temperatures to form geopolymer ceramics, these systems can encapsulate radionuclides such as Cs and Sr within crystalline phases like tobermorite or xonotlite, enhancing long-term stability and leach resistance [30].

The mechanisms of heavy metal stabilization within geopolymers can be summarized as follows [12,31]:

1. **Encapsulation**: Heavy metals are physically trapped within the geopolymer matrix during the polymerization process, limiting their mobility and reducing bioavailability.
2. **Ionic Interactions**: Strong ionic interactions between heavy metals and the geopolymer framework led to the formation of stable chemical complexes, enhancing immobilization.
3. **Precipitation**: The high pH environment created during geopolymerization promotes the precipitation of metal hydroxides, which can coalesce with the geopolymer structure, reducing the solubility of heavy metals.
4. **Formation of Crystalline Phases**: The amorphous nature of geopolymers allows the formation of new crystalline phases that incorporate heavy metals, providing an additional level of stabilization.

Improper disposal of galvanic sludge can lead to severe contamination of soil and water resources, exacerbating the challenges posed by heavy metal pollution. In this context, galvanic sludge—hazardous waste generated from electroplating and metal-finishing industries—poses significant environmental risks due to its high concentrations of heavy metals, including chromium (Cr), nickel (Ni), and iron (Fe). The heavy metals present in galvanic sludge are notoriously toxic and persistent in the environment, necessitating effective stabilization methods to mitigate their harmful effects [32]. Recent studies have highlighted the potential for incorporating industrial wastes, such as galvanic sludge, into geopolymer matrices to immobilize heavy metals and reduce associated environmental risks [33]. By integrating these contaminants within the geopolymer structure, the leaching of toxic metals can be significantly reduced, rendering the waste safer for long-term disposal and preventing further environmental degradation.

The authors have already patented a geopolymer formulation for encapsulating chromium-bearing liquors and other waste products [34], proving that metakaolin-based formulations can immobilize complex waste containing cations, anions, and macromolecules. The absence of literature on the feasibility of stabilizing electroplating sludge in a geopolymeric matrix prompted this study, which focuses on real industrial waste containing high concentrations of Cr and Ni (approximately 270,000 and 140,000 ppm, respectively). This type of waste is particularly complex due to the presence of chromium in cationic (Cr^3+^) and anionic (CrO_4_^2−^) forms, yet the chemistry of its precipitation process resembles that of an alkaline geopolymer paste.

The current study aims to develop a metakaolin-based geopolymer using sodium hydroxide and sodium silicate as activators, incorporating galvanic sludge—a heavy metal-rich industrial waste—to evaluate its potential for immobilizing hazardous metals. The research investigates factors affecting the cold consolidation of the geopolymeric pastes such as molarity and time of preparation of activating solutions, sequence of experimental steps, amount of waste etc. The characterization was carried out employing advanced techniques, such as X-ray diffraction (XRD), Fourier-transform infrared spectroscopy (FT-IR), and environmental scanning electron microscopy (ESEM), to investigate the microstructure and chemical interactions within the geopolymer matrix. Compressive strength tests and water stability assessments were performed to gauge the mechanical performance and durability of the developed geopolymer. Leaching tests were conducted to evaluate heavy metal retention to assess the geopolymer’s effectiveness in immobilizing hazardous materials.

Moreover, the study seeks to understand the formation of the geopolymer and the influence of adding galvanic sludge either before or after the alkaline activation of metakaolin. The formulation of the activating solution was examined by preparing it 24 h prior to geopolymer formation, as well as on the same day, to observe any variations in performance and stability. Different concentrations of sodium hydroxide (NaOH) at 8 and 10 M will be used alongside varying percentages of galvanic sludge added to the mixture.

## 2. Materials and Methods

### 2.1. Raw Materials and Their Characterization

In this study, we utilized ARGICAL^TM^ M1000 metakaolin (MK), sourced from Imerys, Paris, France. According to the manufacturer’s data, the chemical composition of this MK is as follows in Table 1.

Additionally, we examined galvanic sludge (DE), a by-product of industrial metal surface treatment effluents. After drying, DE exhibited a weight reduction of approximately 78%, primarily due to moisture loss. To better understand the composition of DE, the total metal content was determined through sample digestion and subsequent inductively coupled plasma optical emission spectrometry (ICP-OES) and X-Ray fluorescence (XRF) analysis. The sludge was dried by heating on a plate for 24 h at 60 °C and then digested according to the standard procedure outlined in D.Lgs. 152/06, art. 127, using reverse aqua regia (4 mL HNO_3_ + 1 mL HCl). The digested samples were analyzed for metal content using ICP-OES with the iCAP^TM^ PRO ICP-OES system (Thermo Fisher Scientific, Waltham, MA, USA). Cross standard solutions were used to validate the calibration curve. Samples outside the calibration range were suitably diluted to fall within the calibration. Table 2 summarizes the metal concentrations in the galvanic sludge (DE), where Cr, Ni, and Fe were the most abundant elements, present at 26.22%, 13.26%, and 2.85%, respectively. The analysis was also repeated using ZETIUM XRF (Malvern Panalytical, Malvern, Worcestershire, UK) by the “Omniam” method (Table 3). Metal concentrations were derived from the oxide forms reported in the XRF data and showed good agreement with those obtained by combined acid digestion followed by ICP analysis. In addition, the XRF analysis allowed the determination of sulfates, chlorides, and phosphates, which was not possible by the analysis of the digested sample due to the acid attack. A NaOH solution was prepared by dissolving laboratory-grade granules (96 wt%, Sigma Aldrich, Italy) in deionized water to achieve a concentration of 8 and 10 M. Additionally, a commercial sodium silicate solution (Ingessil, Verona, Italy) with a SiO_2_/Na_2_O molar ratio of 3.0, containing 26.50 wt% SiO_2_ and 8.70 wt% Na_2_O, with a pH of 11.7 and a density of 1.368 g/cm^3^ at 20 °C, was used in the formulation of the geopolymers.

### 2.2. Geopolymer Mixture Preparation

**Figure 1:**
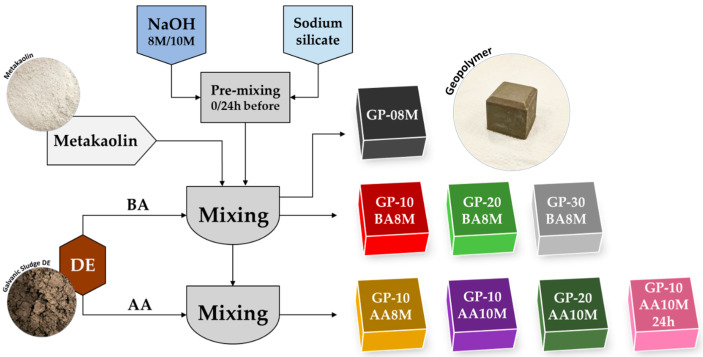
Flowchart showing the synthesis process of all the samples.

In this study, the reference geopolymer (GP-08M), previously optimized in earlier research [35], was prepared by mechanically mixing dry metakaolin powder with an alkaline activating solution composed of 8 M NaOH and Na-silicate. Subsequently, increasing amounts (10, 20, and 30 wt%) of galvanic sludge (DE), calculated with respect to the total mass of metakaolin + DE, were introduced into the fresh mixture. These formulations were denoted as GP-10BA8M, GP-20BA8M, and GP-30BA8M, respectively (Table 4). The acronym BA (“Before Activation”) indicates that DE was mixed with metakaolin simultaneously with the addition of the activating solution. In contrast, in the AA (“After Activation”) formulations, DE was added only after the metakaolin had already been activated by the alkaline solution. A schematic overview of the process is provided in Figure 1.

In two of the formulations (GP-10AA10M and GP-20AA10M), the NaOH concentration was increased to 10 M instead of 8 M, while maintaining a constant Na_2_O content in the activating solution. In the formulation (GP-10AA10M24h), the activating solution (10 M NaOH and Na-silicate) was prepared 24 h in advance at room temperature using magnetic stirring in Teflon^TM^ beakers.

**Figure 2:**
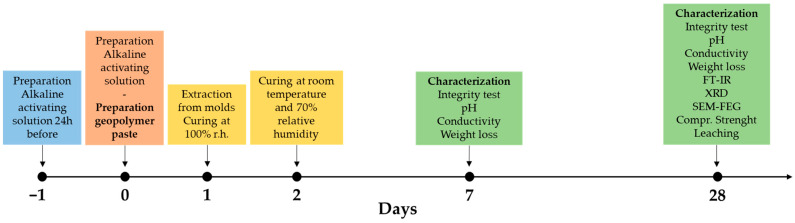
Research plan on a timeline where the different colors correspond to different steps.

All samples were produced using a planetary mixer (Aucma 1400W, Aucma Co., Ltd., Qingdao, China). The fresh paste was cast into 25 × 25 × 25 mm^3^ silicone molds, and air bubbles were removed using a vibrating table. The specimens were then placed in sealed containers at 100% relative humidity and ambient temperature for 24 h, after which they were demolded. Characterizations were carried out on the samples at 7 and 28 days from the date of preparation (Figure 2).

### 2.3. Characterization of Geopolymeric Samples

Given the inherent variability of our waste, the actual metal content in each geopolymer formulation was first verified by digestion followed by ICP analysis. The characterization schedule is shown in Figure 2. The hardened specimens were tested after 7 and 28 days of curing at room temperature in contact with air (approximately 70% relative humidity). First, the degree of geopolymer reticulation was evaluated (Section 2.3.2, Section 2.3.3 and Section 2.3.4). Subsequently, the microstructure and the physical, chemical, and mechanical properties of the specimens were investigated (Section 2.3.5 and Section 2.3.6). Finally, preliminary leaching tests were carried out in accordance with the applicable standards (Section 2.3.7).

#### 2.3.1. Chemical Analysis of Geopolymers

Consolidated geopolymeric materials, after crushing, were digested using a microwave-assisted digestion system (ultraWAVE from Milestone, Sorisole (BG), Italy) with inverse aqua regia (4 mL HNO_3_ + 1 mL HCl) to determine their metal content. The analysis was carried out by ICP-OES in accordance with UNI EN 13656:2004 [36], UNI EN 13657:2004 [37], and UNI EN ISO 11885:2009 [38].

#### 2.3.2. Chemical Stability in Water

In order to assess how the geopolymer paste containing DE consolidated over time, its chemical stability under aqueous conditions was evaluated after 7 and 28 days of curing using an integrity test described in the literature [39] and was determined quantitatively by measuring the weight loss. Deionized water was added at a solid-to-water mass ratio of 1:100, with each dense specimen weighing between 2 and 3 g. After 24 h of immersion, the samples were removed from the water and then dried in acetone for 3 h, followed by a further 3 h of solvent evaporation at room temperature, after which their weights were recorded.

In addition, to evaluate the release of ions from the solid into the water the pH and ionic conductivity (IC) of the eluate were monitored for 48 h in a test similar to UNI EN 12457-2:2004 [40]. See Section 2.3.7 for more details. The test was conducted at a solid-to-liquid ratio of 1:10.

#### 2.3.3. FT-IR Characterization

To assess the extent of geopolymerization, FT-IR spectroscopy (Prestige21 Shimadzu spectrophotometer, Shimadzu Italia s.r.l., Milan, Italy, equipped with a deuterated triglycine sulfate detector and KBr windows) was carried out on geopolymers containing 0/10/20/30 wt% of DE introduced both before (BA) and after (AA) alkaline activation. The spectra were acquired within the 3800–400 cm^−1^ range, at a resolution of 2 cm^−1^, over 60 scans. Pellets composed of 2 mg of sample and 198 mg of KBr were used for the measurements. Subsequent data analysis was performed using Origin 9.1 software.

#### 2.3.4. XRD Characterization

XRD measurements were carried out to identify the crystalline phases within the geopolymer systems, including the formulation containing 10/20/30 wt% DE. The diffractometer was operated at 40 kV and 40 mA with Cu Kα radiation (Ni filter). Diffraction patterns were collected using an X’Celerator detector (X’Pert PRO, PANalytical, Malvern Panalytical Ltd., Malvern, UK) in the 2θ range of 5–70°, with a step size of 0.02°, a count time of 3 s, and a slit width of 10. Phase identification was performed by comparing the experimental peaks with reference patterns in DIFFRAC EVA plus V7 software (Bruker, Milan, Italy, 2005 PDF2).

#### 2.3.5. SEM Observations

A SEM (ESEM-Quanta200, FEI company, Hillsboro, OR, USA) scanning electron microscope equipped with energy dispersive spectroscopy (EDS), was used to investigate the microstructural features of samples cured for 28 days. The analysis aimed at monitoring changes in the amorphous geopolymeric matrix as well as at detecting any unreacted DE or aluminosilicate particles. For SEM observations, a thin gold layer was sputtered onto the freshly fractured surfaces of the specimens.

#### 2.3.6. Compressive Strength

Following a 28-day curing period, the compressive strength of the cubic specimens was determined using an Instron 5567 Universal Testing Machine, Norwood, MA, USA. A maximum load of 30 kN was applied incrementally at a crosshead speed of 1 mm/min.

#### 2.3.7. Leaching Test

The leaching test was carried out according to UNI EN 12457–2:2004 after 28 days of curing. Deionized water was added as a leaching agent to the crushed material—of which at least 95% (by mass) had a particle size of less than 4 mm—maintaining a liquid/solid ratio (L/S) of 10 L/kg ± 2%. After 24 ± 1 h of rotation in an overhead shaker (Rotax 6.8, VELP Scientifica, Usmate, MB, Italy) the suspension was filtered using a 0.45 µm membrane filter. The quantification of soluble metal ions was performed by ICP-OES; anionic species were determined by ion chromatography using an EcoIC system (Metrohm, Herisau, Switzerland).

## 3. Results and Discussion

### 3.1. Quantification of Regulated Metals

In order to determine the real concentration of regulated metals (under Directive (EU) 2018/850/Italy, D. Lgs 121/2020, Annex IV) inside the various geopolymer samples, these ones were digested according to the method described in Section 2.3.1. These results are presented in Table 5, where significant discrepancies in the concentrations of specific metals can be immediately observed when compared to the values expected based on the amount of sludge incorporated into the geopolymers. Despite the different values measured in all the samples except GP-10AA10M24h, the Cr/Ni ratio is consistently around 2 (ranging from 1.9 to 2.4), as can be seen in Table 2 and Table 3, which report on the chemical analysis of the sludge. The Cr/Ni ratio was determined to be 1.98 by ICP and 1.93 by XRF. This suggests that the issue lies in the amount of sludge that has been dissolved, but that the dissolved sludge is chemically consistent. The amounts detected for all the other metals/metalloids (Ba, Cu, Pb and Sb) are consistent across all samples.

### 3.2. Chemical Stability in Water

**Figure 3:**
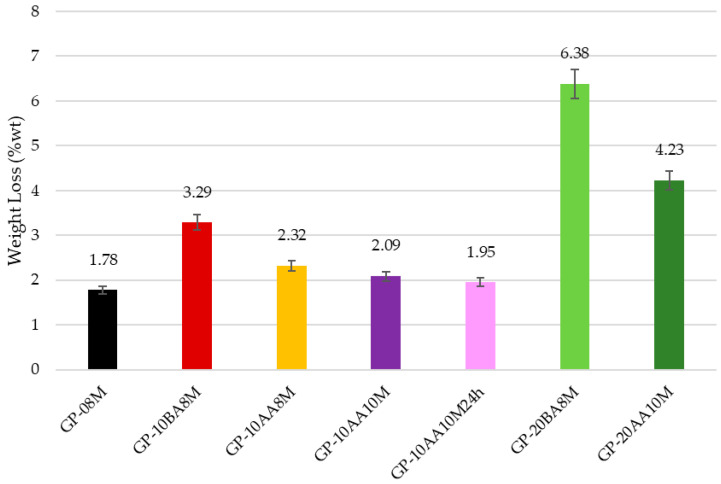
Comparison of the weight loss (WL) at 28 days of all samples after immersion in water. GP-08M is reported as the reference sample. (Error of about 10% was quantified by measuring a large number of samples).

To evaluate the consolidation of geopolymer paste in the presence of DE, all samples, after aging for 28 days, were immersed in deionized water for 24 h with a 1:100 S/W ratio [39]. Firstly, a visual evaluation was performed on the integrity of the samples after the immersion for evaluating stability. Only the sample GP-30BA8M exhibited structural damage after immersion, while all the others maintained their original shape. Secondly, to have a quantitative evaluation, weight loss (WL) was measured on the samples that succeeded in the Integrity test, and the results are shown in Figure 3.

**Figure 4:**
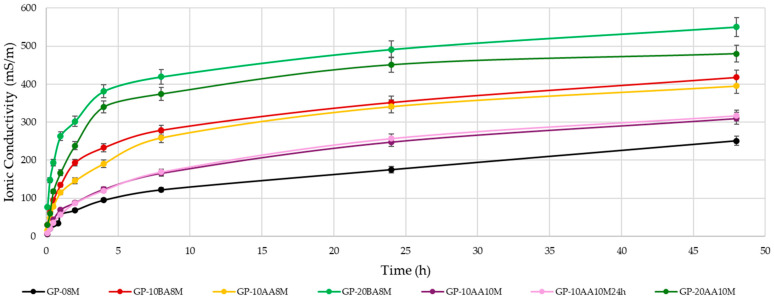
Ionic conductivity of the water after immersion of each sample aged 28 days. (Error of about 9% was quantified by measuring a large number of samples).

It can be seen that the weight loss increases with the percentage of DE added into the paste. An increase in the concentration of the soda solution from 8 M to 10 M positively reduces the WL, reaching values similar to those obtained with the standard formulation of GP-08M (1.78% for reference sample vs. 2.09 and 1.95% for 10 M solution). This behavior confirms that the higher concentration of sodium ions in the activating solution 10 M with respect to the 8 M does not lead to higher conductivity, as they are linked to the [AlO_4_]^−^ species. The presence of Al(III) in four-fold coordination indicates efficient reticulation of the disordered metakaolin aluminosilicate structure [3]. Also, the addition of DE after alkaline activation of MK (AA), seems to affect the WL values less, as shown by the comparison between GP-10BA8M and GP-10AA8M. However, a decrease from 3.29% to 2.32% is still evident. Figure 4 shows a comprehensive representation of the ionic conductivity values of the eluate after immersion in deionized water of the samples with a 1:10 S/W ratio. This test provides information on the total ions present in water resulting from the immersion of the geopolymer, providing insights into the degree of geopolymerization. The ions that most significantly contribute to an increase of conductivity are those with higher ionic mobility, such as Na^+^, OH^−^, and other first- or second-grade ions. In all samples, the pH increases rapidly within the first few minutes, stabilizing above 10 [41]. In contrast, conductivity values exhibit a slower rise. These data demonstrate that increasing the amount of DE from 10 to 20% by weight with respect to metakaolin significantly increases the ion release in solution, demonstrating accordance. This finding is consistent with the considerations made on the WL results and shows that adding DE negatively affects the formation of the geopolymeric structure. Furthermore, the increase in NaOH concentration and the addition of the waste after alkaline activation (AA), rather than before (BA), reduce the ion release, thus confirming the observations made for the WL. Consequently, it can be concluded that using a 10 M NaOH solution and incorporating the waste after alkaline activation leads to a more stabilized structure where geopolymerization occurred.

### 3.3. FT-IR Characterization

**Figure 5:**
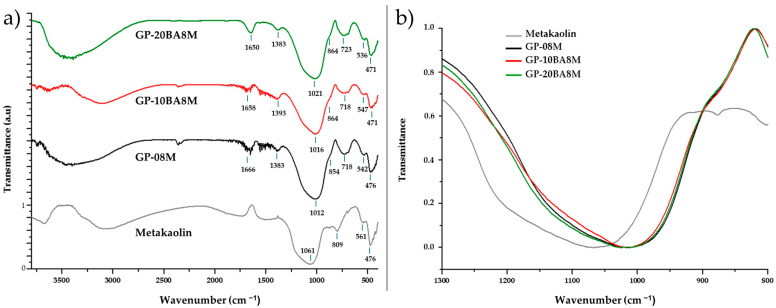
FT-IR spectra of metakaolin and geopolymers containing various percentages of DE (GP0, GP-10BA8M, GP-20BA8M). Shown in (**a**) the spectra acquired between 400 and 3800 cm^−1^, and in (**b**) the magnified view of the band associated with the asymmetric stretching of the Si–O–T bond, normalized for comparison.

In this study, the FT-IR spectra of metakaolin and geopolymers containing varying percentages of DE waste (GP0, GP-10BA8M, and GP-20BA8M) were analyzed to investigate their structural characteristics and assess the impact of DE waste incorporation within the geopolymeric matrix, revealing notable structural changes that occur during the geopolymerization process, as shown in Figure 5. The metakaolin spectrum displays characteristic bands at 1061 cm^−1^, 809 cm^−1^, and 561 cm^−1^, which are attributed to the asymmetric stretching of Si-O-T (T = Si, Al), Si-O stretching corresponding to the presence of quartz, and Al-O stretching of Al in six-fold coordination, respectively [42,43,44,45]. These bands signify the presence of the silicate structure and silanol groups typical of the raw material prior to geopolymerization. Upon activation and curing, the FT-IR spectra of the geopolymers (GP0, GP-10BA8M and GP-20BA8M) reveal distinct shifts, indicating the formation of the geopolymer network [35]. The presence of water is observed in all the geopolymer spectra, with bands around 1650 cm^−1^, corresponding to the bending vibrations of water molecules (H–O–H) [46,47]. This water is likely derived from residues of the activating solution, water generated during the polycondensation reaction, or coordination water surrounding Na^+^ ions within the geopolymeric structure [35]. Notably, the Si–O–T stretching vibration at 1061 cm^−1^ in metakaolin shifts to lower wavenumbers (around 1012 cm^−1^ in GP0, 1016 cm^−1^ in GP-10BA8M, and 1021 cm^−1^ in GP-20BA8M), indicating alterations in the bonding environment as SiO/Al–O bonds are formed, suggesting an increase in Si–O–Al bonds, which are characteristic of geopolymeric structures [42,48,49]. It can be observed that as the percentage of DE waste increases within the geopolymeric matrix, the shift in the Si-O-T stretching absorption becomes less pronounced compared to pure metakaolin, indicating that DE waste does not hinder the formation of the typical three-dimensional geopolymer network. The incorporation of Al into the Si–O–Si network introduces Na^+^ ions to maintain charge balance, thus reducing the amount of sodium leached when the material is immersed in water (Figure 4) [50,51,52]. The reference sample shows, indeed, the lower conductivity value. Additionally, a shoulder around 860 cm^−1^ is observed, which is assigned to the Si–O–non-bridging oxygen (NBO) stretching mode, with one NBO involved per SiO_4_ tetrahedron (Q3 groups, requiring three bridging O and one NBO per SiO_4_ tetrahedron) [53]. Furthermore, the appearance of new bands around 723 cm^−1^, 536 cm^−1^, and 471 cm^−1^ can be attributed to the bending vibration of tetra-coordinated Al(IV)-O-Si, Al-O-Si bending and stretching vibrations, and the Si–O-H bending mode, respectively, all of which support the development of the geopolymer network [54,55,56]. The bending vibration of tetra-coordinated Al(IV)-O-Si observed in the FT-IR spectra of the geopolymers indicates the formation of aluminosilicate networks, as reported by Sitarz et al. [57,58,59,60,61]. The geopolymer samples also display bands around 1360 cm^−1^, which suggest carbonate incorporation due to the carbonation reaction between the alkalis in the geopolymer (mainly Na^+^) and atmospheric CO_2_ [60,61,62]. The carbonate phases are in small amounts because no evidence in the following XRD spectra is shown. These findings underscore the significant structural transformations occurring during geopolymerization, including the dissolution of metakaolin, the formation of the aluminosilicate gel, and the evolution of Si–O–T bonds, ultimately leading to the establishment of a robust geopolymeric matrix.

### 3.4. XRD Characterization

**Figure 6:**
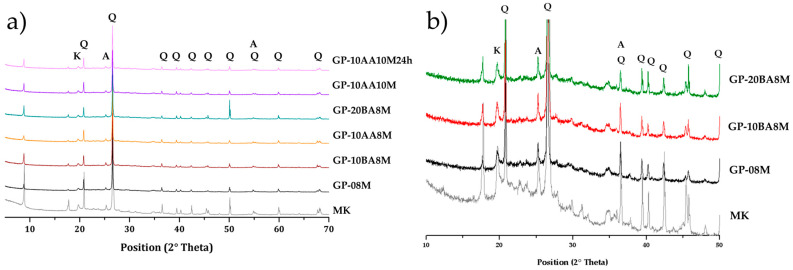
XRD diffractogram of (**a**) Geopolymer samples bearing DE compared with metakaolin (MK) powder and MK-based geopolymer GP0; (**b**) Enlargement of the XRD pattern for samples containing various percentages of DE (GP-10BA8M and GP-20BA8M) compared to MK powder and MK-based geopolymer GP0 in the 10–50° 2θ range.

Mineralogical analysis was conducted on all the samples (Figure 6). In the diffraction pattern of GP0 (Figure 6a), a diffuse reflection is observed, representing the typical broad band of the amorphous aluminosilicate structure. Additionally, sharper peaks corresponding to anatase (A), kaolinite (K) and alpha-quartz (Q) are evident. The diffraction patterns for all the samples are similar, displaying these characteristic features. All geopolymer samples show diffuse reflections typical of an amorphous aluminosilicate network centered around 26–28° in 2θ, with a slight shift toward higher values compared to the pristine metakaolin (Figure 6b) [63,64,65,66,67]. In geopolymeric samples, the peak of kaolinite decreases with respect to metakaolin. Figure 6b presents and compares the enlarged diffractogram in the amorphous region for two samples containing different percentages of DE (GP-10BA8M and GP-20BA8M) with the reference geopolymer (GP0) and the pristine metakaolin. This is to verify whether adding higher percentages of DE has any mineralogical influence. It is evident that the amorphous geopolymer halo between 20 and 35° 2θ remains unaltered, showing no significant shift due to the addition of DE.

The XRD analysis serves as a qualitative method to define the 3D aluminosilicate network of the geopolymer. In this case, the three patterns are nearly identical, indicating that no significant mineralogical changes can be attributed to the presence of DE in the geopolymer matrix.

### 3.5. SEM Observations

**Figure 7:**
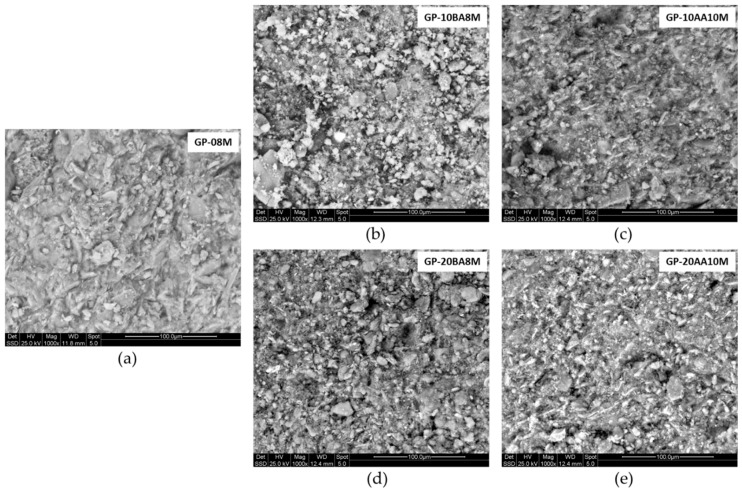
ESEM images of (**a**) GP-08M, (**b**) GP-10BA8M, (**c**) GP-10AA10M, (**d**) GP-20BA8M, (**e**) GP-20AA10M.

This analysis was performed to evaluate the sample’s microstructure and the distribution of certain elements within the material matrix. By analyzing Figure 7, it can be observed that in the fresh fracture of the material, the geopolymeric gel appears homogeneous, with no visible cracks or macropores [68,69]. Also, EDS analysis confirms that Cr, selected as a reference element due to its high concentration in the waste, exhibits a homogeneous distribution, ranging between 0.50% and 0.70%. Therefore, it can be stated that at the microstructural level, a slight deterioration is observed following the addition of DE sludge compared to the GP-08M reference sample, with granules not fully reacted. However, when the sludge is added after the alkaline activation (AA) step and NaOH 10 M is used, the structure becomes significantly more compact, reaching a level comparable to the reference sample.

### 3.6. Compressive Strength

**Figure 8:**
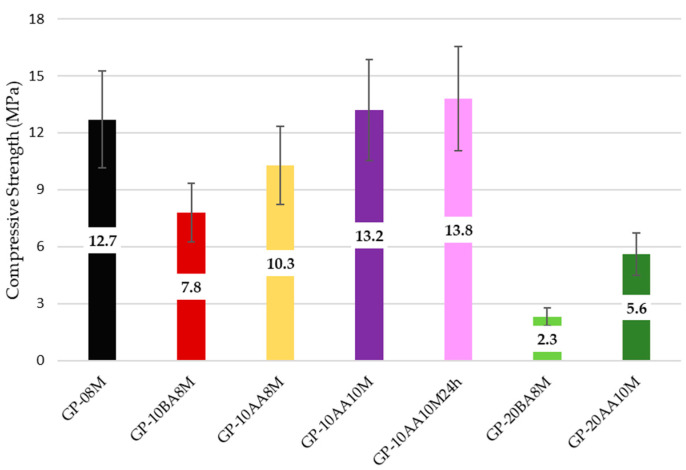
Comparison of compressive strength as a function of the parameters affecting the consolidation process. Samples have been aged for 28 days.

The compressive strength tests were conducted after 28 days to assess whether the obtained geopolymers are self-supporting for potential storage. The results should be considered as indicative, as they were not obtained from a thorough investigation. However, it can be observed that these preliminary data are consistent with the observations made on the mechanical stability of the geopolymers used for stabilization/solidification. In fact, the compressive strength improves with increasing NaOH concentration and when the waste is incorporated after alkaline activation of MK (AA), while it decreases significantly when the sludge content is increased to 20% relative to MK (Figure 8). This behavior is likely due to sludge granules that were not fully incorporated into the geopolymer matrix, as can be seen in the SEM images presented in Section 3.5. These granules disrupt the continuity of the geopolymeric network, causing local structural weakening [70].

Compressive strength can be considered as a three-dimensional overall reticulation index that reflects the effectiveness of the synthesis process. Accordingly, higher compressive strength corresponds to improved chemical properties of the geopolymeric material, as reported in Section 3.2.

### 3.7. Leaching Test

As shown in Table 6, metals such as Cr and Ni exceed the regulatory limits for landfilling as hazardous waste (Implementation of Directive (EU) 2018/850/Italy, D. Lgs 121/2020, Annex IV) [71,72,73]. However, it is important to evaluate the immobilization rate in order to compare the samples among each other by normalizing for the actual heavy metals content within each sample (see Section 3.1), and to assess the material’s performance in metal entrapment, independently of the amount of waste introduced. The high fixation efficiency values, notwithstanding the exceeding of regulatory limits, demonstrates that material effectively immobilizes and stabilizes a significant amount of Cr. The extremely high initial concentration of this element in the waste results in residual levels that still surpass regulatory limits. The immobilization % indicates the proportion of metal effectively retained within the geopolymer, and was calculated as follows:

$$Immobilization\;\left(\%\right)=\frac{C_{G}-C_{L}}{C_{G}}\cdot 100$$

where C_L_ is the content of heavy metal leached out from the geopolymer samples, C_G_ (see Table 5) is the total content of this metal immobilized in the geopolymer sample.

**Figure 9:**
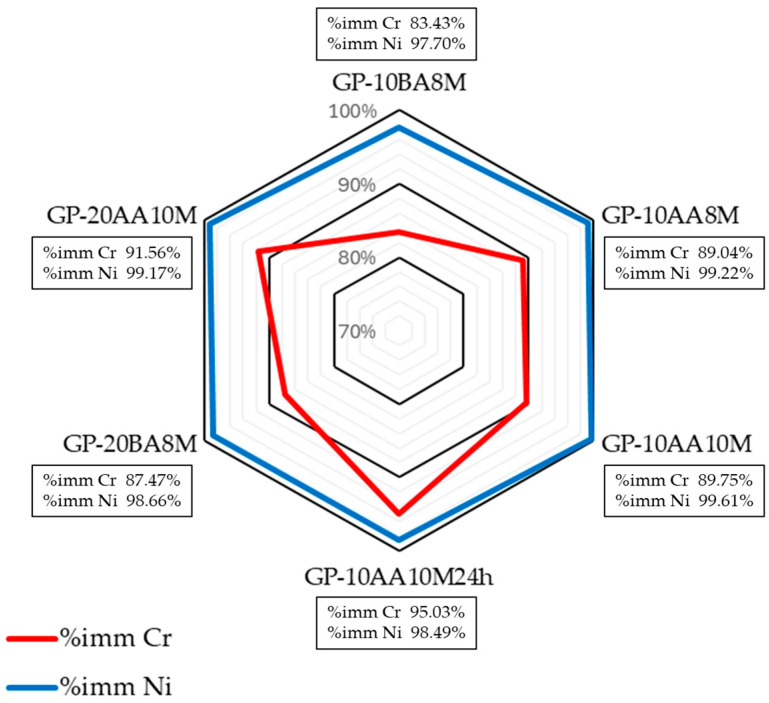
Immobilization % of Cr and Ni in the hardened geopolymers as a function of the parameters affecting the preparation process.

It can be seen in Figure 9 that immobilization rates remain remarkably high, with values above 83.43% for Cr and 97.70% for Ni across all formulations. It was found that the factors affecting immobilization are more evident for Cr than for Ni, whose immobilization percentages are very high. In particular, it was observed that preparing the mixture by adding sludge after metakaolin activation increased Cr immobilization from 83% to 89%. Similarly, preparing the activating solution 24 h before mixing the sludge and geopolymer increased the percentage from 89 to 95.

The difference in the percentage immobilization of Cr and Ni is due to the fact that Ni is present as a cation, while Cr is present as both a cation (Cr^3+^) and an anion (Cr_2_O_7_^2−^) in the geopolymer. Heavy metals in the form of cations can exchange the alkali ions (Na^+^) in the geopolymer matrix and balance the negatively charged Cr_2_O_7_^2−^ tetrahedron. However, the charge neutralization effect does not work between the AsO_4_^3−^ (or, in our case, Cr_2_O_7_^2−^) and [AlO_4_]^−^ tetrahedron, meaning the anions cannot be immobilized as effectively as the cations, as Ji et al. [17] have already observed.

Further, it can be seen that in formulation GP-10BA8M in which the DE sludge was inserted before the alkaline activator (BA), the retention values are generally lower compared to GP-10AA8M formulation, containing the same amount of sludge but added after activation (AA). This suggests that the incorporation of the sludge after the alkaline activation of the metakaolin leads to a more effective encapsulation of heavy metals within the geopolymer network, without any addition of accelerators, which are Na_2_S, NH_4_Cl, FeSO_4_, nor Portland cement, as reported in the literature [70]. Furthermore, in the GP-10AA10M24h sample, where the activating solution of NaOH and sodium silicate was prepared 24 h in advance, the immobilization of Cr seems to be more efficient (increasing from 89.75% to 95.03%). This improvement is probably due to a better homogenization of the two components, resulting in a more effective activation of the aluminosilicate matrix.

In conclusion, the data obtained confirm that the geopolymeric matrix studied is capable of effectively immobilizing both Cr and Ni, even at high concentrations resulting from the addition of galvanic sludge. However, the sludge incorporation strategy (BA vs. AA) and the operational details of the activation process (such as solution pre-preparation) can significantly affect the overall immobilization efficiency.

## 4. Conclusions

The sludge used in this study is a real waste material and therefore exhibits inherent variability, as evidenced by the data obtained from the digested samples, which differ from the theoretical values of the original formulation. Using both direct methods, such as FT-IR and XRD, and indirect methods, including leaching tests, weight loss measurements, ionic conductivity, and compressive strength analysis, it was found that the addition of sludge up to 20% *w*/*w* relative to metakaolin (MK) does not hinder the geopolymerization, affecting only slightly the cross-linking of the geopolymer matrix as shown by SEM analysis. Furthermore, the data consistently indicate that the addition of sludge is a critical factor influencing the material’s final properties, and it should be carried out after the activation (AA) of MK with the alkaline activating solution. The activating solution also plays a significant role in the cross-linking process, as demonstrated by both weight loss and ionic conductivity analyses—two independent methods that confirmed the effectiveness of a NaOH 10 M solution when premixed with the silicate solution 24 h in advance. These observations are supported by SEM analysis which confirms that homogeneous gel is formed in sample AA, activated by NaOH 10 M, while undissolved particles and a lesser amount of gel is shown in samples containing 20 wt% with activation BA and NaOH 10 M. Compressive strength values show the same trend with lower values for samples containing 20 wt% of sludge, BA procedure and NaOH 8 M.

The most critical metals are Cr and Ni, which are present in the dry waste at 27 and 14% by weight, respectively. In this study, they were incorporated into the geopolymers at concentrations up to 13,285 mg/kg for chromium and up to 6943 mg/kg for nickel. Although the leaching values exceed the regulatory limits for these elements, the immobilization percentages are remarkably high, reaching values above 99% for Ni and peaking at 95% for Cr. The difference in the percentage immobilization of Cr and Ni is due to the fact that Ni is present as a cation, while Cr is present as both a cation (Cr^3+^) and an anion (Cr_2_O_7_^2−^) in the geopolymer.

Future studies will focus on the incorporation of stabilizing agents, such as chelating, adsorbing, and reducing agents, capable of improving the retention of heavy metals, and amphoteric elements in the form of cations and anions, without compromising the structural stability of the geopolymers, which have demonstrated the ability to incorporate up to 20% waste content.

## Figures and Tables

**Table 1:** Composition of MK (^a^: loss of ignition at 1000 °C).

SiO_2_	Al_2_O_3_	Fe_2_O_3_	TiO_2_	Na_2_O + K_2_O	CaO + MgO	LOI ^a^
55 wt%	40 wt%	1.4 wt%	1.5 wt%	0.8 wt%	0.3 wt%	1 wt%

**Table 2:** Chemical composition of dried DE determined by ICP-OES from digested samples.

Element (wt%)	DE	Element (ppm)	DE	Element (ppm)	DE
Cr	26.22	Zn	524	Tl	48
Ni	13.26	Mn	426	Sb	41
Fe	2.85	Bi	275	Co	19
Na	1.91	K	152	Ba	13
Ca	1.01	Al	130	Mo	5
Mg	0.59	As	111	Ag	1
Cu	0.59	Pb	95		

**Table 3:** Chemical composition of dried DE determined by XRF.

Element (wt%)	DE	Element (ppm)	DE	Compound (wt%)	DE
Cr	27.38	Zn	583	SO_3_	3.63
Ni	14.19	Sn	445	Cl	2.02
Fe	3.27	Al	278	P_2_O_5_	0.15
Na	2.03	Pb	143		
Ca	1.27	Sr	67		
Mg	0.60				
Cu	0.14				

**Table 4:** Geopolymer formulations (^a^: L/S = liquid [L] (Na-silicate + NaOH) to solid [kg] (MK + DE) ratio; ^b^: MK/DE Metakaolin to DE mass ratio; ^c^: Mass percentage of DE related to the total mass of the fresh paste).

Sample Name	MK [g]	NaOH 8/10 M [g]	Na-Silicate [g]	DE [g]	L/S ^a^ [L/kg]	MK/DE ^b^ [wt/wt]	DE ^c^ [%]
GP-08M	100	38	41	/	0.60	/	/
GP-10BA8M	90	38	41	10	0.60	9.0	5.59
GP-10AA8M	90	38	41	10	0.60	9.0	5.59
GP-20BA8M	80	38	41	20	0.60	4.0	11.17
GP-30BA8M	70	38	41	30	0.60	2.5	16.76
GP-10AA10M	90	31	41	10	0.54	9.0	5.81
GP-10AA10M24h	90	31	41	10	0.54	9.0	5.81
GP-20AA10M	80	31	41	20	0.54	4.0	11.63

**Table 5:** Total effective content of metal in the geopolymers samples. LOD refers to the limit of detection, while LOQ refers to the limit of quantification. Error related to the procedure is estimated at 15%.

Metals	GP-08M	GP-10BA8M	GP-10AA8M	GP-10AA10M	GP-10AA10M24h	GP-20BA8M	GP-20AA10M
	C_G_ (mg/kg)	C_G_ (mg/kg)	C_G_ (mg/kg)	C_G_ (mg/kg)	C_G_ (mg/kg)	C_G_ (mg/kg)	C_G_ (mg/kg)
As	<LOD	<LOQ	<LOD	<LOD	<LOD	<LOQ	<LOD
Ba	<LOD	207.42	181.86	196.36	150.06	235.01	189.24
Cd	<LOD	<LOD	<LOD	<LOD	<LOD	<LOD	<LOD
Cr	0.011	4251.21	4877.51	5915.57	1043.00	7867.72	13,285.01
Cu	<LOD	29.58	28.72	32.83	171.14	46.56	78.60
Mo	<LOD	<LOD	<LOD	<LOD	<LOD	<LOD	<LOD
Ni	<LOD	2107.08	2253.80	2478.94	1506.51	3780.29	6943.35
Pb	<LOD	63.45	<LOQ	41.34	48.83	87.56	49.00
Sb	<LOD	44.30	42.47	92.10	85.58	76.03	89.09
Se	<LOD	<LOQ	<LOQ	<LOD	17.03	<LOQ	21.13
Zn	0.034	<LOQ	<LOQ	46.85	934.01	<LOQ	91.13

**Table 6:** Leaching concentrations of the samples, expressed in mg/L, were prepared using a ratio L/S = 10 L/kg compared to the limits of the Directive (EU) 2018/850. Error related to the procedure is estimated at 15%.

Metals and Anions	GP-08M	GP-10BA8M	GP-10AA8M	GP-10AA10M	GP-10AA10M24h	GP-20BA8M	GP-20AA10M	Inert	Non-Hazardous	Hazardous
	C_L_ (mg/L)	C_L_ (mg/L)	C_L_ (mg/L)	C_L_ (mg/L)	C_L_ (mg/L)	C_L_ (mg/L)	C_L_ (mg/L)			
As	0.245	0.258	0.210	0.238	0.176	0.259	0.233	0.05	0.2	2.5
Ba	0.03	0.03	0.02	0.02	0.02	0.02	0.03	2	10	30
Cd	<LOD	<LOD	<LOD	<LOD	<LOD	<LOD	<LOD	0.004	0.1	0.5
Cr	0.08	70.44	53.45	60.63	5.18	98.60	112.06	0.05	1	7
Cu	0.01	0.05	0.02	0.01	0.40	0.06	0.07	0.2	5	10
Mo	0.01	0.02	0.01	0.01	0.05	0.02	0.01	0.05	1	3
Ni	<LOD	4.85	1.75	0.97	2.27	5.05	5.75	0.04	1	4
Pb	0.019	0.01	0.01	0.02	0.04	0.01	0.02	0.05	1	5
Sb	0.016	0.003	<LOD	<LOD	0.014	0.003	<LOD	0.006	0.07	0.5
Se	<LOD	0.024	<LOD	0.016	0.040	0.022	<LOD	0.01	0.05	0.7
Zn	0.03	<LOD	<LOD	<LOD	1.03	<LOD	<LOD	0.4	5	20
F^−^	<LOD	4.77	4.68	5.27	1.24	5.5	6.94	1	15	50
Cl^−^	<LOD	37.0	38.5	39.0	4.72	62.9	98.3	80	2500	2500
SO_4_^2−^	<LOD	94.9	93.7	96.6	63.4	138	152	100	5000	5000

## Data Availability

The original contributions presented in this study are included in the article. Further inquiries can be directed to the corresponding author.
